# Study protocol for the Champaign-Urbana population study

**DOI:** 10.3389/fnimg.2026.1728970

**Published:** 2026-02-10

**Authors:** Paul B. Camacho, Aaron T. Anderson, Rong Guo, Yuhui Chai, Sina Tafti, Ian Hall, Dominika M. Pindus, Chris Lockwood, Paul M. Arnold, Sheeba Arnold-Anteraper, Zhi-Pei Liang, Hacene Serrai, Andrew G. Webb, Bansari Upadhyay, Diane Beck, Mark D. Whiting, Bruce M. Damon, Tracey M. Wszalek, Brad P. Sutton

**Affiliations:** 1 Beckman Institute for Advanced Science and Technology, University of Illinois at Urbana-Champaign, Urbana, IL, United States; 2 Carle-Illinois Advanced Imaging Center, Carle Health, Urbana IL and University of Illinois at Urbana-Champaign, Urbana, IL, United States; 3 Siemens Medical Solutions USA, Inc., Malvern, PA, United States; 4 Institute for Applied Life Sciences, University of Massachusetts Amherst, Amherst, MA, United States; 5 The Grainger College of Engineering, Office of the Associate Dean for Research, University of Illinois Urbana-Champaign, Urbana, IL, United States; 6 Department of Neurosurgery, Loyola University School of Medicine, Chicago, IL, United States; 7 Carle-Illinois College of Medicine, Urbana, IL, United States; 8 Advanced Imaging Research Center, University of Texas Southwestern Medical Center, Dallas, TX, United States; 9 The Grainger College of Engineering, Electrical and Computer Engineering, University of Illinois Urbana-Champaign, Urbana, IL, United States; 10 Stephens Family Clinical Research Institute, Carle Health, Urbana, IL, United States; 11 CJ Gorter Center for High Field MRI, Leiden University Medical Center, Leiden, Netherlands; 12 Department of Psychology, University of Illinois at Urbana-Champaign, Urbana, IL, United States; 13 Department of Bioengineering, The Grainger College of Engineering, University of Illinois Urbana-Champaign, Urbana, IL, United States; 14 Department of Biomedical Engineering, Vanderbilt University, Nashville, TN, United States; 15 Department of Radiology and Radiological Sciences, Vanderbilt University Medicine Center, Nashville, TN, United States

**Keywords:** 7 Tesla, cognition, diffusion weighted (DW) MRI, functional MRI, MR spectroscopic imaging, neuroimaging, population study, ultra-high field

## Abstract

Superior signal-to-noise ratio, enhanced and novel forms of contrast, and improved spectral resolution made possible by 7 Tesla (7 T) magnetic resonance imaging (MRI) offer great promise for both neuroimaging research and clinical practice. To characterize these gains, it is essential to acquire structural, functional, and biochemical 7 T MRI data from a large sample of adults. The Champaign Urbana Population Study (CUPS) will collect and publish a database of 7 T MRI data, including raw MRI data, from a cohort of up to 200 adults. Here, we describe the study design and provide example images from the initial round of data collection for CUPS.

## 1 Introduction

With the development of 7 T magnetic resonance imaging (MRI) systems over the past 20 years (Uğurbil, 2018) and the commercialization of FDA-cleared models in the past 8 years, many neuroimaging researchers and clinicians are excited by 7 T MRI’s potential for using higher spatial resolution and enhanced or alternative contrast to discover new structure/function relationships in the brain. In a prominent effort for using 7 T MRI to expand our understanding of brain connectivity, the Human Connectome Project (HCP) 7 T subset improved on the original 3 T HCP protocol (Van Essen et al., 2013) to collect higher spatial resolution anatomical, diffusion-weighted imaging (DWI) (Vu et al., 2015), and functional MRI (fMRI) (Vu et al., 2017) data. This study represents the largest number of individuals to be sampled in a published 7 T MRI dataset to date (target *n =* 200).

Several other 7 T MRI initiatives have made progress on intensive within-individual sampled task fMRI (Gonzalez-Castillo et al., 2015; Allen et al., 2022; Hanke et al., 2014), harmonization of quantitative susceptibility mapping (QSM) (Rua et al., 2020), subcortical fMRI (Groot et al., 2024), faster anatomical tissue segmentation (Svanera et al., 2021), sub-millimeter diffusion mapping (Wang et al., 2021), and quantitative T1 and T2* mapping (Tardif et al., 2016; Alkemade et al., 2020). These studies and their novel datasets have consistently shown that significant gains in our understanding of the brain are available with well-tuned protocols and advances in image acquisition and processing that enable researchers to address the technical challenges that accompany the increases in signal. These challenges include increased specific absorption rate, wave-interference effects on B1 + field homogeneity, and B0 inhomogeneity (Bernstein et al., 2006; Yang et al., 2002; Stockmann and Wald, 2018).

The improvement in sensitivity at 7 T compared to 3 T have been shown in standard research imaging approaches. Chu and colleagues recently investigated that morphometry of anatomical images from participants scanned at both 3 T and 7 T. This analysis showed age-related differences in the same number of regions with *n =* 117 participants at 7 T versus *n =* 350 participants at 3 T (Chu et al., 2025). Similar increases in statistical significance and sensitivity to smaller effects have been shown at 7 T compared to 3 T in task fMRI (Torrisi et al., 2018). Significant features found in 7 T images can also be used to inform lower field strength MRI applications, allowing for clinical applications outside of 7 T research centers. By training machine learning models with paired data from higher field strengths and low field strengths, the quality of MRI data collected at as low as 64mT can be increased (Iglesias et al., 2022; Islam et al., 2023). Deep learning models trained on large MRI datasets have shown improved detection ability for age-related brain atrophy at 55 mT (Man et al., 2023; Lau et al., 2023).

Previous medium- to large-scale 7 T MRI studies have focused on benefits from improved resolution in structural T1-weighted and T2-weighted scanning (Keuken et al., 2014; Isaacs et al., 2020; De Ciantis et al., 2016), the increased contrast to noise ratio in functional MRI (Welvaert and Rosseel, 2013; Vizioli et al., 2021; Liu et al., 2022), and higher spatial resolution in DWI (Wu et al., 2016; Kleinnijenhuis et al., 2015; Ma et al., 2023). However, there are additional contrasts available that will also benefit from the increased field strength that have not been explored in previous large scale studies. For example, magnetic resonance spectroscopy (MRS) and MR spectroscopic imaging (MRSI) detect brain metabolites and neurotransmitters. Thus, MRS and MRSI benefit both from higher spatial resolution enabled by improved signal-to-noise ratio (SNR) and from increased spectral resolution to characterize and differentiate biochemicals in the brain. At the same time as the field strength is increasing, new MRS approaches (such as SPectroscopic Imaging by exploiting spatiospectral CorrElation, SPICE) (Guo et al., 2021; Lam et al., 2020) are being fine-tuned that leverage spatiotemporal correlations in the high dimensionality data to further improve the resolution and speed of metabolic imaging. To realize the true potential of high field MRI, we can couple increased field strength and SPICE acquisition and reconstruction to examine metabolic profiles of substructures of the brain in relation to healthy variation, age, and disease.

An additional new imaging technique that has shown strong potential for increasing our sensitivity to individual differences in brain structure and function is magnetic resonance elastography (MRE) which provides a measurement of tissue mechanical properties (Kruse et al., 2008). For example, in healthy young adult males, variations in hippocampal viscoelasticity partially mediated the relationship between aerobic fitness and performance on a relational memory task (Schwarb et al., 2016, 2017; Hiscox et al., 2020). Hippocampal stiffness at 3 T shows some potential as a biomarker for temporal lobe epilepsy (Huesmann et al., 2020). This method relies on high SNR in the phase signal in the imaging data. While ensuring that higher static field inhomogeneities do not corrupt the signals, then spatial resolution can be increased while maintaining a sufficient phase SNR at 7 T, enabling MRE to probe finer scale structural and functional properties of the brain.

Susceptibility-weighted imaging (SWI) leverages the increased sensitivity to magnetic susceptibility differences at 7 T to detect levels of myelin, iron, and calcium within tissues (Spincemaille et al., 2020; Langkammer et al., 2012; Haacke et al., 2004). Using multiple echoes of SWI, QSM yields voxel-wise magnetic susceptibility values (Li and Leigh, 2004; Liu et al., 2011b; Schweser et al., 2011). Clinical applications for QSM include detecting cerebral microbleeds (Perosa et al., 2023) along with cortical and paramagnetic rim lesions in multiple sclerosis (Barletta et al., 2021; Kaunzner et al., 2019; Meaton et al., 2022). Age-related differences in *χ* are seen in subcortical regions, hippocampus, motor and superior frontal regions, as well as the cerebellum (Betts et al., 2016; Madden and Merenstein, 2023; Guevara et al., 2024).

In contrast to studies of specific clinical conditions, population neuroscience seeks to understand how the nervous system changes across a broader range of individuals using a combination of demographic data, behavioral measures, imaging, and other samples (Paus, 2010; Falk et al., 2013; Paus, 2024). Population neuroimaging draws on large sample sizes to study the variability of imaging measures and how these might predict risk of cognitive decline and nervous system disorders (Falk et al., 2013; Vernooij et al., 2016; Hall et al., 2018). Importantly, these studies have less restrictive inclusion criteria and draw from more representative samples to reduce the likelihood of selection bias and capture this variance (Paus, 2010). This epidemiological approach allows researchers to consider the social and environmental effects on the relationship between the brain and behavior.

To build on previous large-cohort 7 T MRI studies (Van Essen et al., 2013; Alkemade et al., 2020), and to incorporate more forms of contrast and quantitative mapping sequences into the neuroimaging protocol, we are conducting the Champaign Urbana Population Study (CUPS). The imaging data collection is accompanied by actigraphy data collection for habitual physical activity and survey data collection for general background; racial, ethnic, and sex demography; hearing; cognitive performance; and personal and family medical history. This dataset will therefore provide a large sample of 7 T and associated data in a manner that represents our local population, producing better generalizability of the findings. As with prior 7 T studies, we also aim to drive the development of 7 T processing tools and resources with a rich set of imaging sequences. These data and methods will be made publicly available.

## 2 Methods and analysis

All methods are approved by the Institutional Review Board (IRB) at Carle Foundation Hospital, to which the University of Illinois at Urbana-Champaign IRB defers on this study. Voluntary informed consent is required for all participants. Participants are compensated $20/h of experimental time.

### 2.1 Design

**Figure 1:**
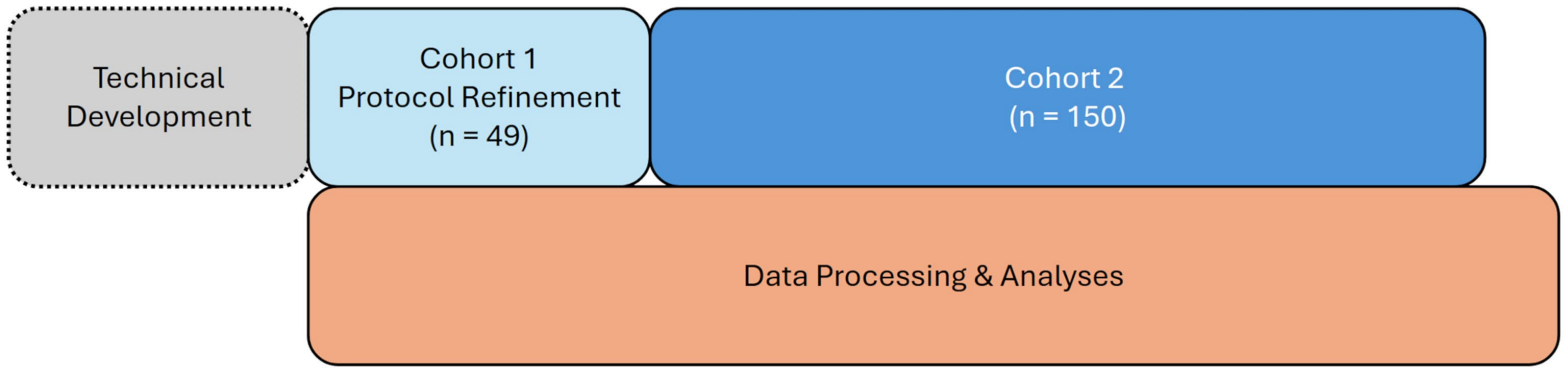
Timeline for CUPS study.

CUPS is an observational study, beginning with an imaging acquisition and processing protocol development Cohort 1 (*n =* 49) and a larger Cohort 2 (*n =* 150, see Figure 1). Non-identifiable data from Cohort 2 will be made publicly available in the Brain Imaging Data Structure (BIDS) (Gorgolewski et al., 2017) through OpenNeuro.

### 2.2 Sampling plan

Enrollment is open to adults aged 18 years or older who are free of 7 T MRI contraindications, and able to provide informed consent. Recruitment mechanisms include word of mouth, flyers, internet (including the CUPS website),^1^ local print/broadcast media, and social media. Participants are screened for eligibility according to the inclusion/exclusion criteria listed in Table 1. Participants with conditions known to show differences in MRI will not be excluded, unless they show diminished decision making capacity. Recruitment goals will be age-stratified to match the local demographics with the following number of participants per age range: 47 in the 18–29 range, 25 in the 30–39 range, 21 in the 40–49 range, 20 in the 50–59 range, 19 in the 60–69 range, 12 in the 70–79 range, and 6 in the 80 + range. We aim to recruit an equal number of male and female participants. Recruitment will also reflect the race and ethnicity demographics of the Champaign County, Illinois area: 64.7% White (non-Hispanic), 11.6% Black or African American (non-Hispanic), 9.36% Asian (non-Hispanic), 3.25% Two Races Excluding Other & Three or More Races (non-Hispanic), 0.59% Two Races including Other (non-Hispanic), 0.36% Other (non-Hispanic), 2.98% Two Races Including Other (Hispanic), 2.38% White (Hispanic), 1.49% Other (Hispanic), 0.245% Black or African American (Hispanic), and 0.194% Two Races Excluding Other & Three or More Races (Hispanic). We also aim to match the local annual household income ranges (40% under $50,000, 31% $50,000 - $100,000, 22% $100,000 – $200,000, 7% over $200 K) and highest level of education achieved (4% no degree, 25% high school, 26% some college, 22% bachelor’s degree, 23% post-graduate). Self-Report Questionnaires & Activity Tracking.

**Table 1:** Inclusion and exclusion criteria at time of study enrollment.

Inclusion criteria at time of study enrollment
Age 18 years or older
Good or corrected vision and hearing
English or Spanish speaking
No current or past diagnosis of mild cognitive impairment or dementias
No MRI contraindications (e.g., metal, or implanted devices in the body)
Willing to share non-identifiable data in a public database

Exclusion criteria at time of study enrollment
Self-reported pregnancy
Diminished decision-making capacity
Physician-diagnosed disorders affecting temperature regulation of the body core or of the head and neck

Diminished capacity is indicated by an inability to complete the study questionnaires, interview, and cognitive assessments, or an inability to provide informed consent and understand the nature and goals of the study. Evidence of impaired decisional capacity is evaluated by study team members per Carle policy. Potential participants are eligible if they do not have conditions that would impair their ability to respond to the measures are requested.

Self-report questionnaire data include: (1) demographic, general health from the Short-Form (SF-36) Survey (Ware and Sherbourne, 1992), and social history; (2) habitual physical activity levels (Sallis et al., 1985); (3) Edinburgh handedness inventory (EDI) (Veale, 2014; Wiberg et al., 2019; Oldfield, 1971); (4) Hearing Handicap Inventory for Adults (HHIA) (Newman et al., 1990). The 7-Day Physical Activity Report quantifies recent habitual physical activity levels (Sallis et al., 1985). The EDI will be used to determine left or right hand dominance in activities of daily living (Veale, 2014; Wiberg et al., 2019; Oldfield, 1971). These data are stored and managed in REDCap (Harris et al., 2009).

Physical activity for Cohort 2 will be tracked using an ActiGraph wGT3X-B (Ametris, Pensacola, Florida, USA) for the initial phase (*n =* 49 participants, data collected for seven consecutive days) and activPAL4 for the remaining participants (*n =* 150 participants, data collected for 14 consecutive days). Accelerometry intensity metrics will include minutes spent in daily physical activity intensities (sedentary, light, moderate and vigorous) using a set of validated cut points (Hildebrand et al., 2014; Migueles et al., 2021), as well as non-cut point-dependent metrics, including average acceleration, intensity gradient (a metric summarizing individual’s daily physical activity intensity profile) (Rowlands et al., 2018) and acceleration (mg) above which an individual’s daily most active minutes are accumulated (Rowlands et al., 2019). ActivPAL measured outcomes will include time spent sitting/lying, standing, number of sit-to-stand and stand-to-sit transitions, and daily steps (O’Brien et al., 2022; Montoye et al., 2022). We note that the ActivPAL has shown differing sensitivity to sedentary versus standing behavior compared to the ActiGraph in some populations (Barboza et al., 2022; Wullems et al., 2024). However, both show agreement in detection of stepping activity (Radtke et al., 2021).

Participants will perform the following tests from the NIH Toolbox Cognition Battery (Denboer et al., 2014; Weintraub et al., 2013): the Flanker Inhibitory Control and Attention Test, the Rey Auditory Verbal Learning Test, the Dimensional Change Card Sort Test, and the Pattern Comparison Processing Speed Test. The purpose of these cognitive assessments is to assess various domains of cognitive ability, such as *executive function* via the Dimensional Change Card Sort Test and the Flanker Inhibitory Control and Attention Test, *memory* via the Rey Auditory Verbal Learning Test, *learning* via the Rey Auditory Verbal Learning Test, *attention* via the Dimensional Change Card Sort Test and the Flanker Inhibitory Control and Attention Test, and *processing speed* via the Pattern Comparison Processing Speed Test. These will be administered to the participant via iPad by a trained study team member using the official NIHToolbox app (Gershon et al., 2013),^2^ under the supervision of co-author TMW.

#### 2.2.1 Neuroimaging data

MRI data will be collected by registered MRI technologists using a single American College of Radiology (ACR)-accredited Siemens 7 T MR system (MAGNETOM Terra, Siemens Healthineers, Erlangen, Germany) at the Carle-Illinois Advanced Imaging Center. Radiofrequency excitation and signal reception use a Nova Medical 8Tx/32Rx (Nova Medical, Inc., Wilmington, MA, USA) parallel transmit head coil operated in circularly polarized (CP) mode. The system undergoes daily quality assurance (QA) procedures, including echo-planar imaging (EPI) stability and ACR phantom testing using FDA-approved coils and weekly EPI stability and Siemens’ customer QA tests using the parallel transmit head coil. The imaging sequences will include several structural, functional, metabolic, and MRE sequences. A magnetization prepared 2 rapid acquisition gradient echoes (MP2RAGE) (Marques et al., 2010) will be used for the T1-weighted structural scan (further details are included in Table 2). Resting-state fMRI data will be collected using the Center for Magnetic Resonance Research (CMRR) multiband sequence (10.7 min, 1.18 s TR, 25 ms TE, 1.6 mm^3^ isotropic voxel size, multiband factor of 5, and iPAT factor of 2), (Moeller et al., 2010; Feinberg et al., 2010; Xu et al., 2013) with phase-encoding direction-flipped field maps (Auerbach et al., 2013). DWI data will also be collected with the CMRR multiband sequence (Auerbach et al., 2013; Setsompop et al., 2012) (1.6 mm^3^ isotropic voxel size, 64 directions at *b* = 1000 *s/mm*^2^ and at *b* = 2000 *s/mm*^2^, 4 *b* = 0 volumes, multiband factor of 4 with iPAT factor of 3) and accompanying phase encode reversed field maps. A T2*-weighted, high resolution gradient echo (G)RE sequence will be used for hippocampal imaging. High resolution spectroscopic mapping will use custom SPICE sequences (metabolite acquisition: 3 mm isotropic voxel size, TR 150 ms, TE 1.4 ms, flip angle 26 degree, matrix size = 78x78x24, vector size = 184; water (T1/T2/QSM/T2*) acquisition: 1 mm isotropic voxel size 55 ms TR, 1.4 ms TE, 3 T1 frames (flip angles: 7, 17, 27 degrees), 3 T2 frames (preparation times: 40, 70, 100 ms), matrix size = 224x216x72; total scan time for all acquisitions = 7 min) (Guo et al., 2019; Guo et al., 2021; Guo et al., 2025). Quantitative Susceptibility Mapping (QSM) will use A Simple Phase Imaging REconstruction method (ASPIRE; Eckstein et al., 2018) phase unwrapping. Tissue mechanical property mapping will be performed using a custom multiband spiral MRE pulse sequence as in Johnson et al. (2013, 2014) with 1.25 mm^3^ isotropic resolution, in-plane parallel imaging factor of 4, multiband excite 2 slices and encode 2 slices, 50 Hz actuation, with 4 time offsets, 78 mT/m encoding gradient. The sensitivity of the MRE sequence is 0.452 rad/*μ*m (Guenthner et al., 2018). Actuation is performed for the MRE using a Resoundant (Rochester Minnesota) and a head pad. The B1 field will be mapped using the dual refocusing echo acquisition mode (DREAM) sequence (Ehses et al., 2019; Nehrke and Börnert, 2012). MRI scan sessions are split into two same-day one-hour parts as needed with a break. When feasible to collect, a FLAIR image will be acquired at 0.7 mm isotropic voxel size, using a T2 SPACE non-selective dual-inversion recovery sequence with TI1/TI2 of 3120/450 ms.

**Table 2:** MRI acquisition parameters for the CUPS 7 Tesla MRI data.

Scan	TR (s)	TE (ms)	Flip angle (degrees)	Voxel size	Number of slices	Other
MP2RAGE	4.53	2.26	4 TI1, 5 TI2	0.75 mm isotropic	240	TI1/2 = 750/2950, GRAPPA acceleration factor = 3, slice partial Fourier = 6/8
Resting-state fMRI	1.18	25	60	1.6 mm isotropic	95	520 time-points
DWI	3.7	89.6	90	1.6 mm isotropic	92	64 directions, b = 1,000, 2000
T2*- weighted	1.12	20	52	0.35×0.35 ×1 mm	56	aligned perpendicular hippocampus
Spectroscopic mapping (metabolite/water)	0.150/0.055	1.4/1.4	26 3 T1 frames 7, 17, 27	3 mm isotropic/1 mm isotropic	72	vector size = 184, matrix size = 78x78x24 matrix size = 224x216x72, 3 T2 frames (TP = 40, 70, 100 ms)
QSM	46	4, 8, 12…40	9	1.0 mm isotropic	144	10 echoes
Tissue stiffness mapping (MRE)	0.160	80	-	1.25 mm isotropic	96	50 Hz encoding, flow compensated gradients, 0.452 rad/*μ*m sensitivity
FLAIR	8.0	264	120	0.7 mm isotropic	224	Non-Sel DIR TI1/2 = 3120/450
B1 DREAM	6.0	1.12, 2.19	60	4.0 mm isotropic	52	

TR, Repetition time; TE, echo time; MP2RAGE, Magnetization prepared rapid acquisition gradient echoes; TI, inversion time; GRAPPA, Generalized autocalibrating partially parallel acquisitions; fMRI, Functional magnetic resonance imaging; DWI, Diffusion-weighted imaging; TP, Time of preparation; QSM, Quantitative susceptibility mapping.

A qualified study team member or an MRI technologist may note an incidental research image observation in a scan of a CUPS participant. If the observation is made by a study team member, they would then alert the CIAIC MRI technologists. The CIAIC MRI technologists will note the randomized participant identification numbers of any studies to be reviewed. These randomized participant identification numbers will be transmitted to the physician for their review. For each participant in this list, the reviewer will review a limited set of images. The anticipated turnaround time for the physician to review and report back on the incidental research observation is about 1 week. If a completed review form has the option “YES” selected for “recommend follow-up with a primary care provider,” then the research participant liaison will be notified. The research participant liaison would then provide the imaging files to the participant and will advise them to contact their primary care physician for further consultation and evaluation. This interaction is guided by a script and cover letter.

### 2.3 Analysis plan

To facilitate reproducible analyses (Poldrack et al., 2017), we developed a software container-based processing pipeline for the MRI modalities collected herein. These are compatible with the BIDS (Gorgolewski et al., 2016) standard at the time of this publication (Camacho et al., 2021; see Code Availability). As part of adapting this pipeline to high-performance computing systems, this pipeline uses internally and externally developed BIDS-Apps (Gorgolewski et al., 2017) converted from Docker images (Merkel et al., 2014) to Singularity/Apptainer images (Kurtzer et al., 2017; Combe et al., 2016). The pipeline steps are run with the Slurm Workload Manager (Yoo et al., 2003) (SchedMD LLC, Lehi, Utah, USA). Specific versions for BIDS-Apps are listed herein and will be updated if serious issues are identified and resolved in later releases.

**Figure 2:**
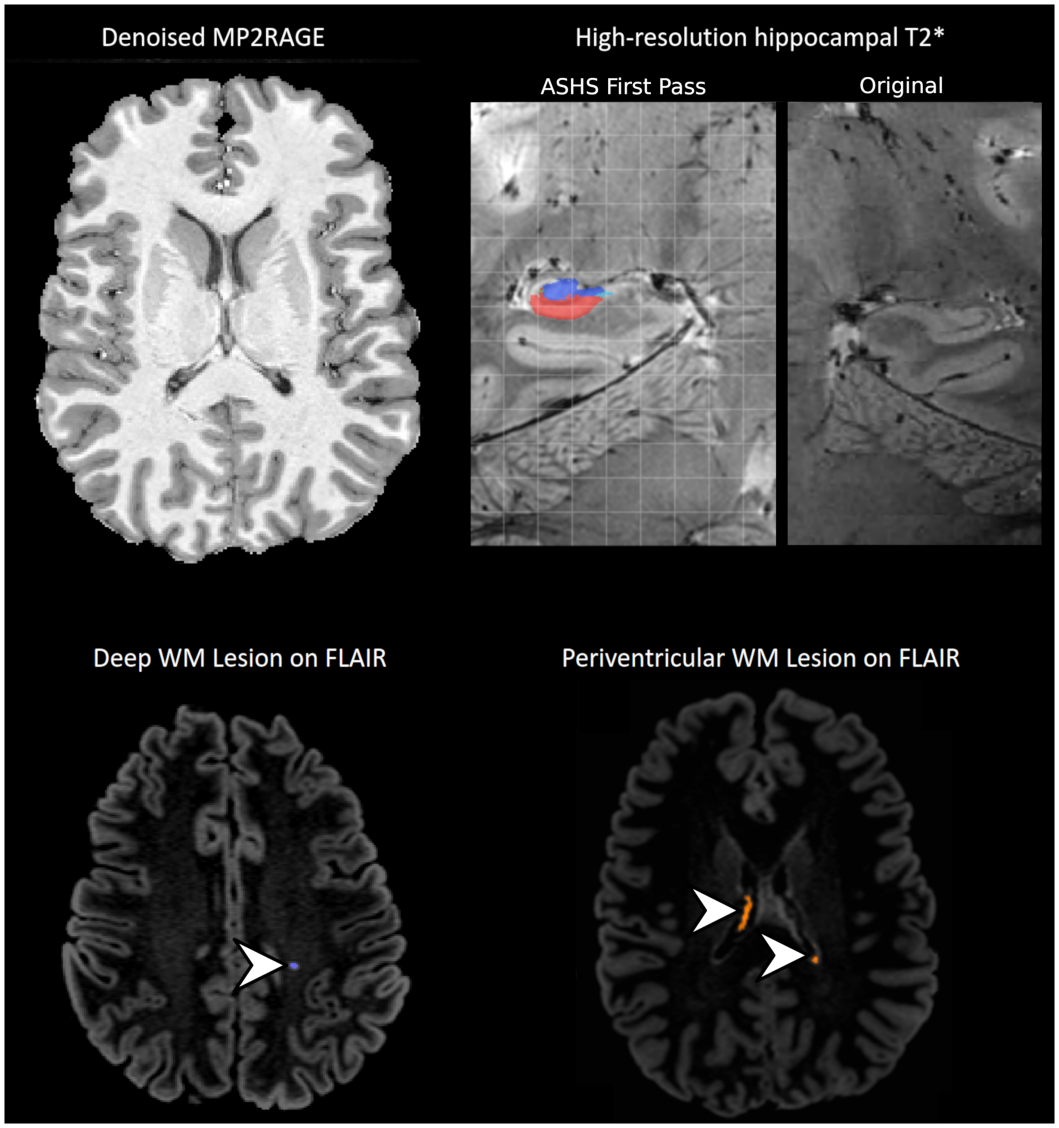
Example anatomical data from one participant in CUPS. Arrows point to lesions detected by Lesion-Mapper-BIDS in the deep white matter and periventricular regions. MP2RAGE: A magnetization prepared 2 rapid acquisition gradient echoes, WM: White matter, FLAIR: Fluid-attenuated inversion recovery.

Preprocessing begins with DICOM to BIDS format NIFTI conversion using HeuDiConv (Li et al., 2016). For better performance in brain extraction, skull-stripping, and registrations, MP2RAGE UNI images are denoised using the LN MP2RAGE DNOISE tool from LAYNII (Huber et al., 2021) – based on a method developed by O’Brien et al. (2013) – with a beta regularization term of 0.4 (see Figure 2).

#### 2.3.1 Anatomical pre-processing

The T1-weighted (T1w) image will be corrected for intensity non-uniformity (INU) with N4BiasFieldCorrection (Tustison et al., 2010), distributed with ANTs 2.3.3 (Avants et al., 2008, RRID:SCR_004757), and used as T1w-reference throughout the workflow. The T1w-reference will then be skull-stripped with a *Nipype* implementation of the antsBrainExtraction.sh workflow (from ANTs), using OASIS30ANTs as target template. Brain tissue segmentation of cerebrospinal fluid (CSF), white-matter (WM) and gray-matter (GM) will be performed on the brain-extracted T1w using fast (FSL 6.0.5.1:57b01774, RRID:SCR_002823, Zhang et al., 2001). Brain surfaces will be reconstructed using recon-all (FreeSurfer 7.3.2, RRID:SCR_001847, Dale et al., 1999), and the previously estimated brain mask will then be refined with a custom variation of the method to reconcile the ANTs-derived and FreeSurfer-derived segmentations of the cortical gray matter using Mindboggle (RRID:SCR_002438, Klein et al., 2017).

Volume-based spatial normalization to two standard spaces (MNI152Nlin2009cAsym, MNI152Nlin6Asym) will be performed through nonlinear registration with antsRegistration (ANTs 2.3.3), using brain-extracted versions of the T1w reference and the T1w template. The following templates were selected for spatial normalization and will be accessed with *TemplateFlow* (23.0.0, Ciric et al., 2022): *ICBM 152 Nonlinear Asymmetrical template version 2009c* [Fonov et al. (2011), RRID:SCR_008796; TemplateFlow ID: MNI152Nlin2009cAsym], *FSL’s MNI ICBM 152 non-linear 6th Generation Asymmetric Average Brain Stereotaxic Registration Model* [Evans et al. (2012), RRID:SCR_002823; TemplateFlow ID: MNI152Nlin6Asym].

#### 2.3.2 Functional pre-processing

**Figure 3:**
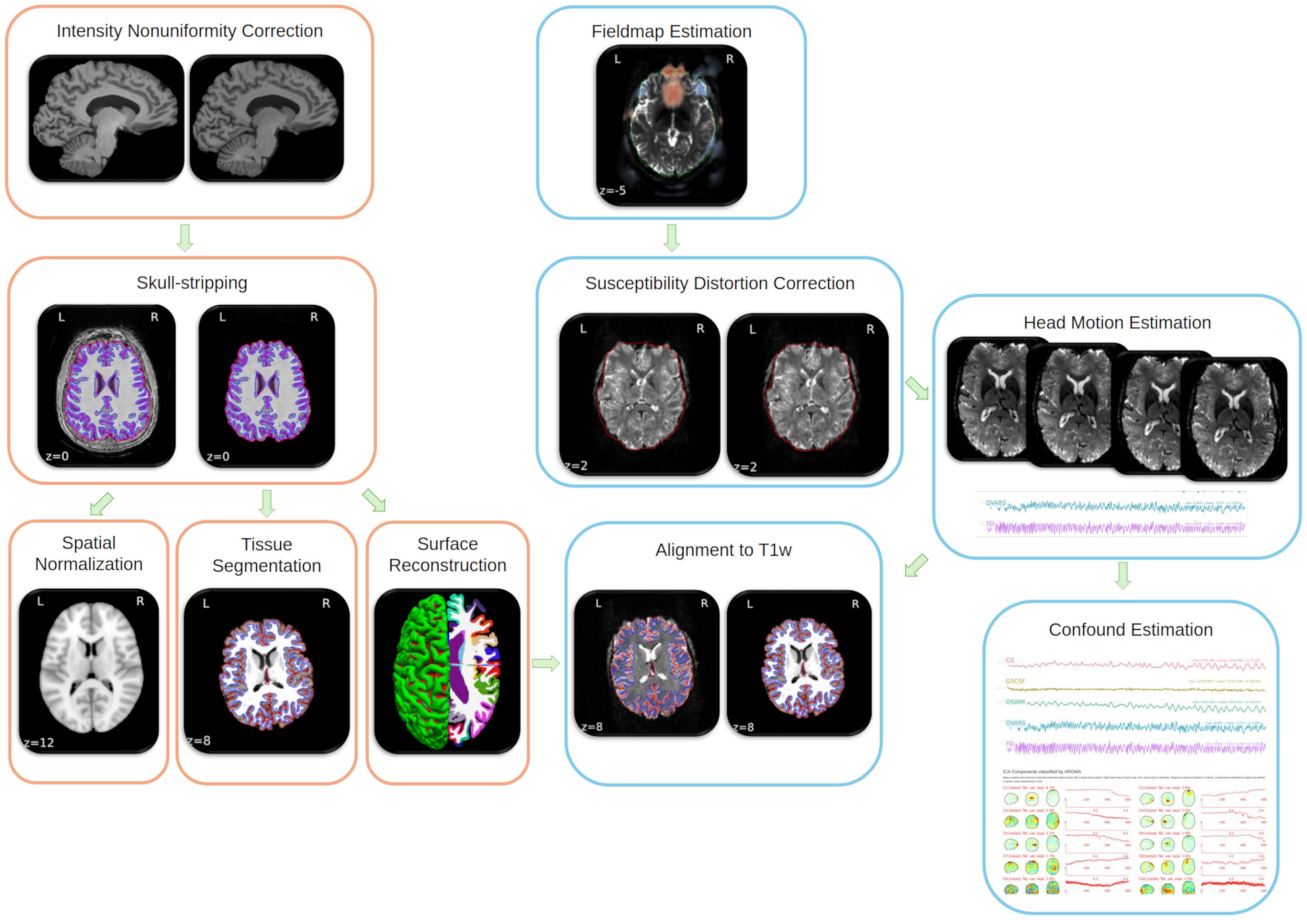
Visual overview of the fMRIPrep workflow. T1w: T1-weighted.

Preprocessing will then be performed using *fMRIPrep* 23.0.2 (Esteban et al., 2018b; Esteban et al., 2018a; RRID:SCR_016216), which is based on *Nipype* 1.8.6 (Gorgolewski et al., 2011; Gorgolewski et al., 2018; RRID:SCR_002502). See Figure 3 for an overview of the workflow. A total of 2 echo-planar imaging (EPI) field maps will be available within the input BIDS structure for each participant at each Session, one for the resting state fMRI scan and one for the diffusion scan. A *B0*-nonuniformity map (or *fieldmap*) will then be estimated based on two (or more) EPI references with topup (Andersson et al., 2003; FSL 6.0.5.1:57b01774). A reference volume and its skull-stripped version will be generated using a custom methodology of *fMRIPrep*. Head-motion parameters with respect to the BOLD reference (transformation matrices, and six corresponding rotation and translation parameters) will be estimated before any spatiotemporal filtering using mcflirt (FSL6.0.5.1:57b01774, Jenkinson et al., 2002). The estimated *fieldmap* will then be aligned with rigid-registration to the target EPI b = 0 image. The field coefficients will then be mapped on to the reference EPI using the transform. BOLD runs will be slice-time corrected to 0.559 s (0.5 of slice acquisition range 0 s-1.12 s) using 3dTshift from AFNI (Cox and Hyde, 1997, RRID:SCR_005927). The BOLD reference will then be co-registered to the T1w reference using bbregister (FreeSurfer) which implements boundary-based registration (Greve and Fischl, 2009). Co-registration will be configured with six degrees of freedom. Several confounding time-series will be calculated based on the *preprocessed BOLD*: framewise displacement (FD), DVARS (Derivative of time-series Root Mean Square of the VARiance over Voxels), and three region-wise global signals. FD will be computed using two formulations following Power [absolute sum of relative motions, Power et al. (2014)] and Jenkinson [relative root mean square displacement between affines, Jenkinson et al., 2002]. FD and DVARS will be calculated for each functional run, both using their implementations in *Nipype* (following the definitions by Power et al., 2014). The three global signals will then be extracted within the CSF, the WM, and the whole-brain masks for regression and signal correction (Jenkinson and Smith, 2001).

Additionally, a set of physiological regressors will be extracted to allow for component-based noise correction (*CompCor*, Behzadi et al., 2007). Principal components will be estimated after high-pass filtering the *preprocessed BOLD* time-series using a discrete cosine filter with 128 s cut-off for the two *CompCor* variants: temporal (tCompCor) and anatomical (aCompCor). tCompCor components will then be calculated from the top 2% variable voxels within the brain mask. For aCompCor, three probabilistic masks (CSF, WM, and combined CSF + WM) will be generated in anatomical space. The implementation differs from that of Behzadi et al. (2007) in that instead of eroding the masks by two pixels in BOLD space, a mask of pixels that likely contain a volume fraction of GM will be subtracted from the aCompCor masks. This mask will be obtained by dilating a GM mask extracted from the FreeSurfer’s *aseg* segmentation, and it will ensure components are not extracted from voxels containing a minimal fraction of GM. Finally, these masks will be resampled into BOLD space and binarized by thresholding at 0.99 (as in the original implementation). Components will also be calculated separately within the WM and CSF masks. For each CompCor decomposition, the *k* components with the largest singular values will be retained, such that the retained components’ time-series will be sufficient to explain 50 percent of variance across the nuisance mask (CSF, WM, combined, or temporal). The remaining components will be dropped from consideration. The head-motion estimates calculated in the correction step will also be placed within the corresponding confounds file. The confound time-series derived from head motion estimates and global signals will be expanded with the inclusion of temporal derivatives and quadratic terms for each (Satterthwaite et al., 2013).

Frames that exceeded a threshold of 0.5 mm FD or 1.5 standardized DVARS will be annotated as motion outliers. Additional nuisance time-series will be calculated using principal components analysis of the signal found within a thin band (*crown*) of voxels around the edge of the brain, as proposed by Patriat et al. (2017). The BOLD time-series will then be resampled into standard space, generating a *preprocessed BOLD run in MNI152Nlin2009cAsym space*. Automatic removal of motion artifacts using independent component analysis (ICA-AROMA, Pruim et al., 2015) will be performed on the *preprocessed BOLD in MNI space* time-series after removal of non-steady state volumes and spatial smoothing with an isotropic, Gaussian kernel of 6 mm FWHM (full-width half-maximum). Corresponding “non-aggressively” denoised runs will be produced after such smoothing. Additionally, the “aggressive” noise-regressors will be collected and placed in the corresponding confounds file. All spatial resamplings will be performed with *a single interpolation step* by composing all the pertinent transformations (i.e., head-motion transform matrices, susceptibility distortion correction when available, and co-registrations to anatomical and output spaces). Gridded (volumetric) resamplings will be performed using antsApplyTransforms (ANTs), configured with Lanczos interpolation to minimize the smoothing effects of other kernels (Lanczos, 1964). Non-gridded (surface) resamplings will be performed using mrivol2surf (FreeSurfer). Many internal operations of *fMRIPrep* use *Nilearn* 0.9.1 (Abraham et al., 2014, RRID:SCR_001362), mostly within the functional processing workflow. For more details of the pipeline, see the section corresponding to workflows in *fMRIPrep*’s documentation.

The above boilerplate text was automatically generated by fMRIPrep and minimally edited for readability. It is released under the CC0 license.

#### 2.3.3 Resting-state functional connectivity post-processing

**Figure 4:**
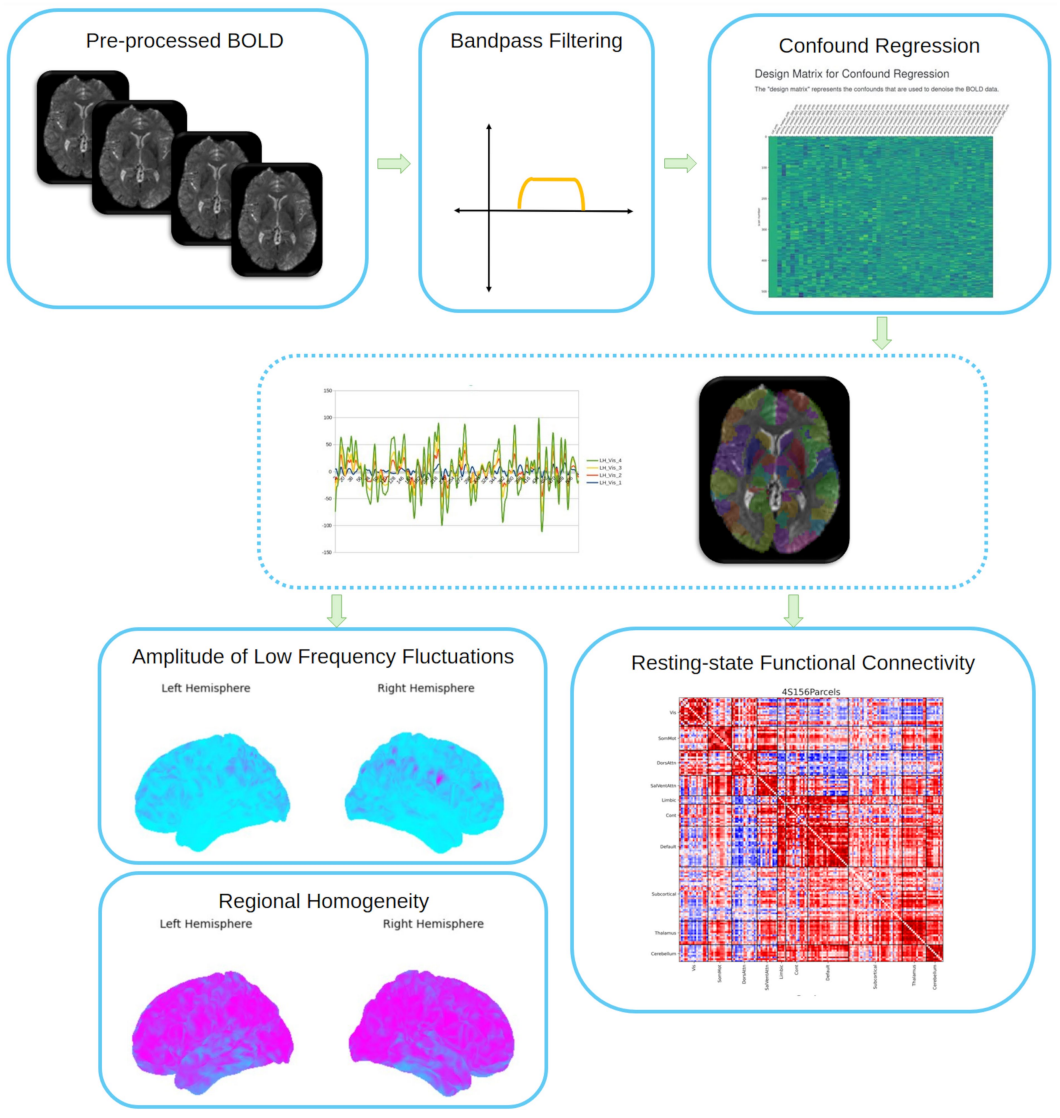
Overview of post-processing in XCP-D. Parcellation shown for resting-state functional connectivity estimation using the 4S156 atlas. BOLD: Blood-oxygen-level dependent fMRI.

The eXtensible Connectivity Pipeline- DCAN (XCP-D) (Ciric et al., 2018; Satterthwaite et al., 2013) will be used to post-process the outputs of *fMRIPrep* version 23.0.2 (Esteban et al., 2018b, RRID:SCR_016216). See Figure 4 for a visual overview of this workflow. XCP-D was built with *Nipype* version 1.8.6 (Gorgolewski et al., 2011, RRID:SCR_002502). Native-space T1w images will be transformed to MNI152Nlin2009cAsym space at 1*mm*^3^ resolution. The six translation and rotation head motion traces will be band-stop filtered to remove signals between 0.2 and 0.3 Hz using a fourth-order notch filter, based on Fair et al. (2020). The Volterra expansion of these filtered motion parameters will then be calculated. Framewise displacement will be calculated from the filtered motion parameters using the formula from Power et al. (2014), with a head radius of 50 mm. Nuisance regressors will be selected according to the ‘aroma’ strategy. AROMA motion-labeled components (Pruim et al., 2015), mean white matter signal, and mean cerebrospinal fluid signal will be selected as nuisance regressors (Ciric et al., 2017; Satterthwaite et al., 2013). AROMA non-motion components (i.e., ones assumed to reflect signal) will be used to account for variance by known signals. Prior to denoising the BOLD data, the nuisance confounds will be orthogonalized with respect to the non-motion components. In this way, the confound regressors will be orthogonalized to produce regressors without variance explained by known signals, so that signal would not be removed from the BOLD data in the later regression. Nuisance regressors will be regressed from the BOLD data using a denoising method based on *Nilearn*’s approach. The timeseries will then be band-pass filtered using a second-order Butterworth filter, in order to retain signals between 0.01–0.08 *Hz*. The same filter will then be applied to the confounds. The resulting time-series will then be denoised using linear regression. The denoised BOLD will be smoothed using *Nilearn* with a Gaussian kernel (*FWHM* = 3.0 *mm*).

The amplitude of low-frequency fluctuation (ALFF) (Zou et al., 2008) will be computed by transforming the mean-centered, standard deviation-normalized, denoised BOLD time-series to the frequency domain. The power spectrum will be computed within the 0.01–0.08 *Hz* frequency band and the mean square root of the power spectrum will be calculated at each voxel to yield voxel-wise ALFF measures. The resulting ALFF values will then be multiplied by the standard deviation of the denoised BOLD time-series to retain the original scaling. The ALFF maps will be smoothed with Nilearn using a Gaussian kernel (*FWHM* = 3.0 *mm*). Regional homogeneity (ReHo) (Jiang and Zuo, 2016) will be computed with neighborhood voxels using *AFNI*’s *3dReHo* (Taylor and Saad, 2013).

Processed functional timeseries will be extracted from the residual BOLD signal with *Nilearn’s NiftiLabelsMasker* for the following atlases: the Schaefer Supplemented with Subcortical Structures (4S) atlas (Schaefer et al., 2018; Pauli et al., 2018; King et al., 2019; Najdenovska et al., 2018; Glasser et al., 2013) at 3 different resolutions (156, 256, 456), the Glasser atlas (Glasser et al., 2016), the Gordon atlas (Gordon et al., 2016), the Tian subcortical atlas (Tian et al., 2020), and the HCP CIFTI subcortical atlas (Glasser et al., 2013). Corresponding pair-wise functional connectivity between all regions will be computed for each atlas, which will be operationalized as the Pearson’s correlation of each parcel’s unsmoothed timeseries. In cases of partial coverage, uncovered voxels (values of all zeros or NaNs) will either be ignored (when the parcel had *>* 50.0% coverage) or will be set to zero (when the parcel had *<* 50.0% coverage). Many internal operations of *XCP-D* use *AFNI* (Cox, 1996; Cox and Hyde, 1997), *ANTS* (Avants et al., 2009), *TemplateFlow* version 24.2.0 (Ciric et al., 2022), *matplotlib* version 3.9.2 (Hunter, 2007), *Nibabel* version 5.2.1 (Brett et al., 2022), *Nilearn* version 0.10.4 (Abraham et al., 2014), *NumPy* version 2.1.1 (Harris et al., 2020), *pybids* version 0.17.1 (Yarkoni et al., 2019), and *scipy* version 1.14.1 (Virtanen et al., 2020). For more details, see the *XCP-D* website.^3^

The above methods description text for the Resting-State Functional Connectivity Post-Processing section was automatically generated by *XCP-D* and minimally edited for readability. It is released under the CC0 license.

#### 2.3.4 Diffusion pre-processing

**Figure 5:**
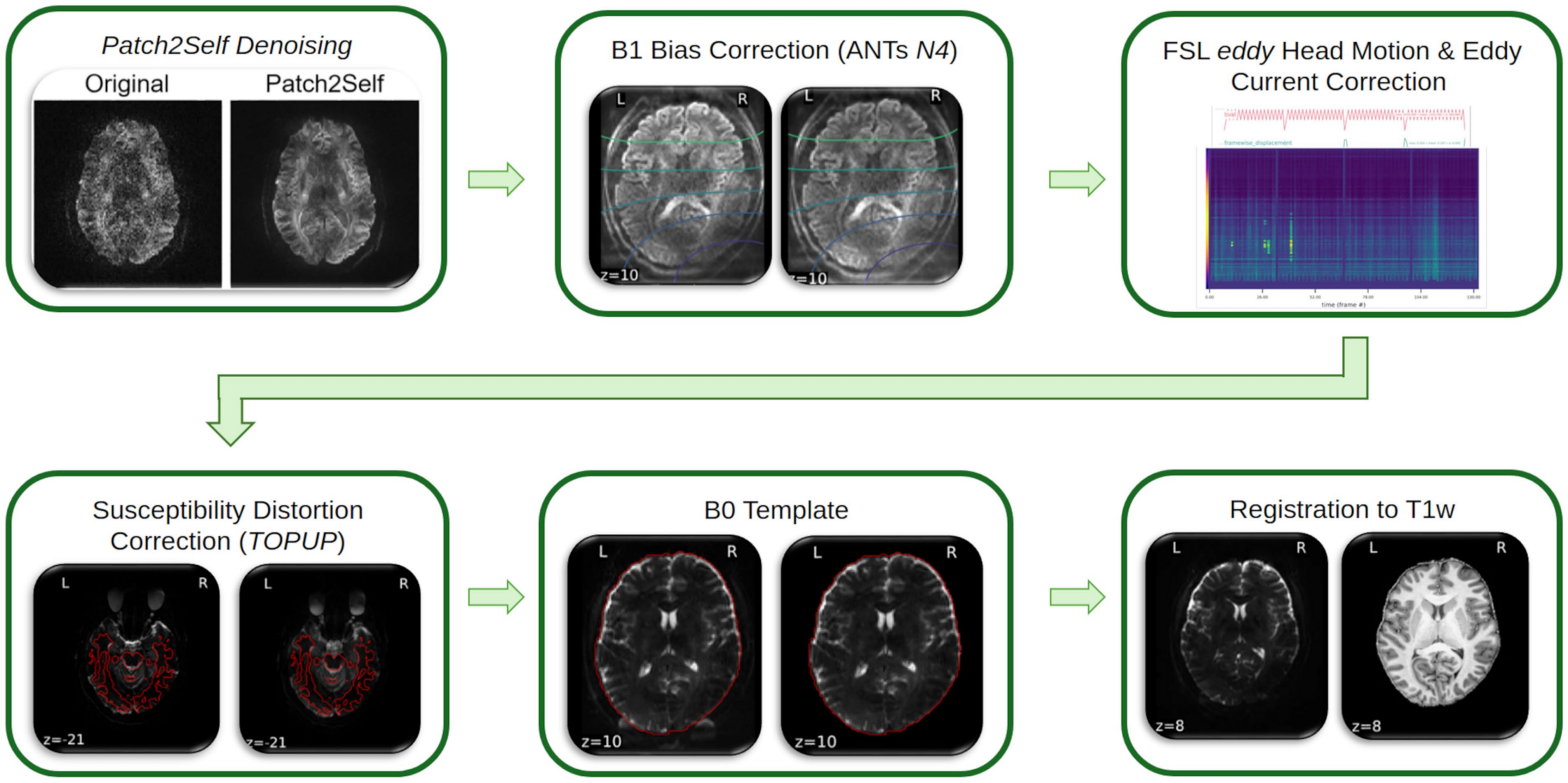
Diffusion pre-processing overview for QSIPrep. ANTs: Advanced normalization tools, B0: *b*-value = 0, T1w: T1-weighted.

Preprocessing will be performed using *QSIPrep* 1.0.0, which is based on *Nipype* 1.9.1 (Gorgolewski et al., 2011; Gorgolewski et al., 2018; RRID:SCR_002502). See Figure 5 for a visual review of this workflow. Any images with a *b*-value less than 100 s/mm^2^ will be treated as a *b* = 0 image. DWI data will be denoised using *DiPy*’s *Patch2Self* algorithm (Garyfallidis et al., 2014; Fadnavis et al., 2020) with an automatically-defined window size. B1 field inhomogeneity will be corrected using dwibiascorrect from Mrtrix3 with the N4 algorithm (Tustison et al., 2010).

FSL (version 6.0.3:b862cdd5)’s eddy will be used for head motion correction and Eddy current correction (Andersson and Sotiropoulos, 2016). *Eddy* will be configured with a *q*-space smoothing factor of 10, a total of five iterations, and 1,000 voxels used to estimate hyperparameters. A linear first level model and a linear second-level model will be used to characterize Eddy current-related spatial distortion. *Q*-space coordinates will be forcefully assigned to shells. We will attempt to separate field offsets from subject movement. Shells are aligned post-eddy. *Eddy*’s outlier replacement will be run (Andersson et al., 2016). Data will be grouped by slice, only including values from slices determined to contain at least 250 intracerebral voxels. Groups deviating by more than four standard deviations from the prediction will have their data replaced with imputed values. Data for the field maps will be collected with reversed phase-encode blips, resulting in pairs of images with distortions going in opposite directions. Here, b = 0 reference images with reversed phase encoding directions will be used along with an equal number of b = 0 images extracted from the DWI scans. From these pairs the susceptibility-induced off-resonance field will be estimated using a method similar to that described in (Andersson et al., 2003). The field maps will ultimately be incorporated into the Eddy current and head motion correction interpolation. Final interpolation will performed using the jac method.

Several confounding time-series will be calculated based on the preprocessed DWI: FD using the implementation in *Nipype* (Power et al., 2014). The head-motion estimates calculated in the correction step will also be placed within the corresponding confounds file. Slice-wise cross correlation will also be calculated. The DWI time-series will be resampled to ACPC, generating a *preprocessed DWI run in ACPC space* with 1.6 mm isotropic voxels. A final DWI to T1w co-registration will be performed in ants Apply Transforms using the rigid transformation from ants Registration of the b = 0 reference image in ACPC space, the pre-processed T1w image, and their respective brain masks.

Many internal operations of *QSIPrep* use *Nilearn* 0.10.1 (Abraham et al., 2014) and *Dipy* 0.18.0 (Garyfallidis et al., 2014). For more details of the pipeline, see the section corresponding to workflows in *QSIPrep*’s documentation.^4^

#### 2.3.5 Diffusion post-processing

**Figure 6:**
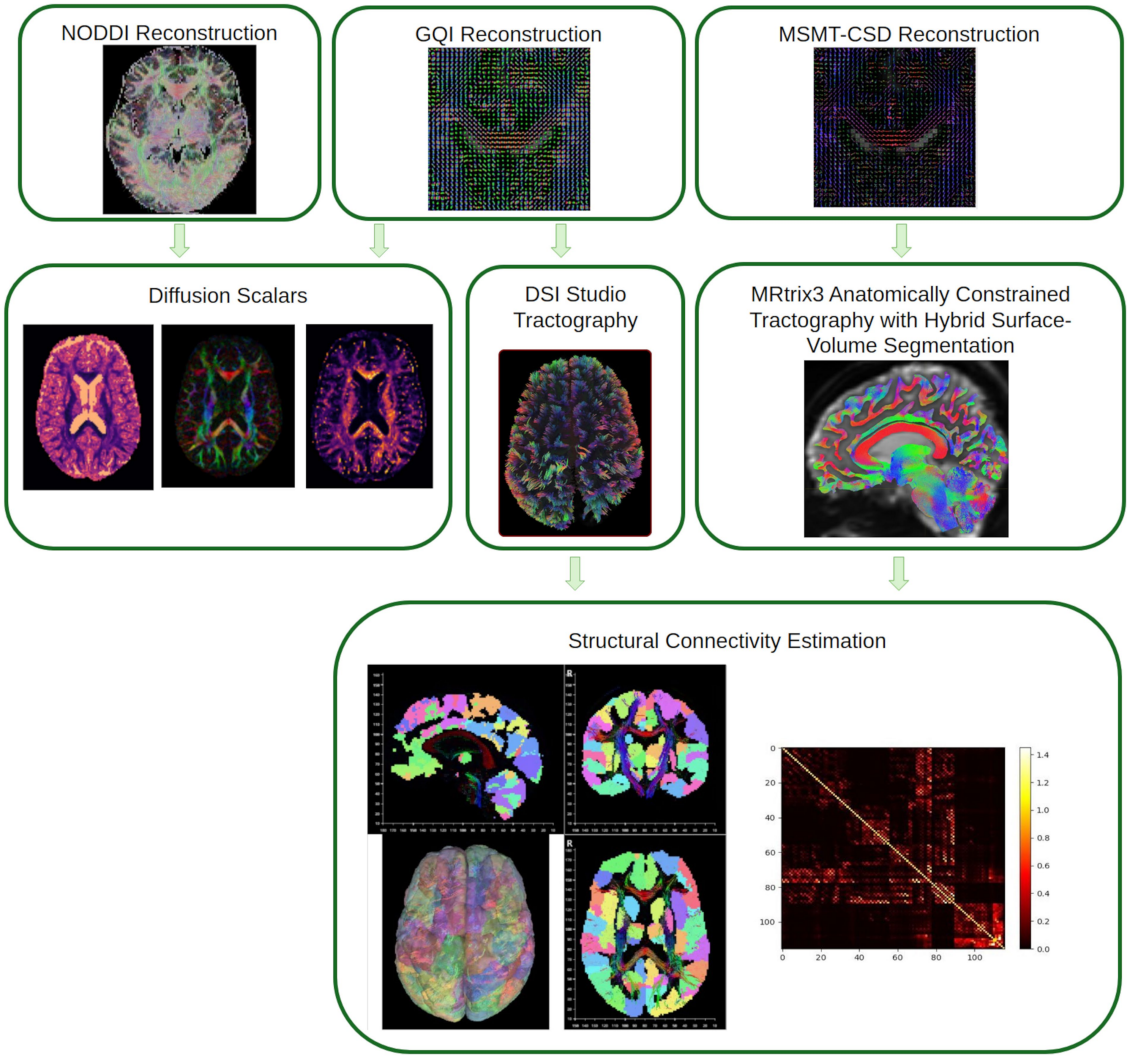
Visual summary of diffusion reconstruction methods used in QSIRecon. Parcellation shown in the structural connectivity estimation using the 4S156 atlas. NODDI: Neurite orientation dispersion and density imaging, GQI: Generalized q-sampling imaging, MSMT-CSD: Multi-shell multi-tissue constrained spherical deconvolution.

T1w-based spatial normalization calculated during preprocessing will be used to map atlases from template space into alignment with DWIs. Brain masks from antsBrainExtraction will be used in all subsequent reconstruction steps. The following atlases will be used in the workflow: the Schaefer Supplemented with Subcortical Structures (4S) atlas (Schaefer et al., 2018; Pauli et al., 2018; King et al., 2019; Najdenovska et al., 2018; Glasser et al., 2013) at 3 different resolutions (156, 256, 456 parcels). Cortical parcellations will be mapped from template space to DWIs using the T1w-based spatial normalization. The following reconstruction workflows are visually summarized in Figure 6.

**Figure 7:**
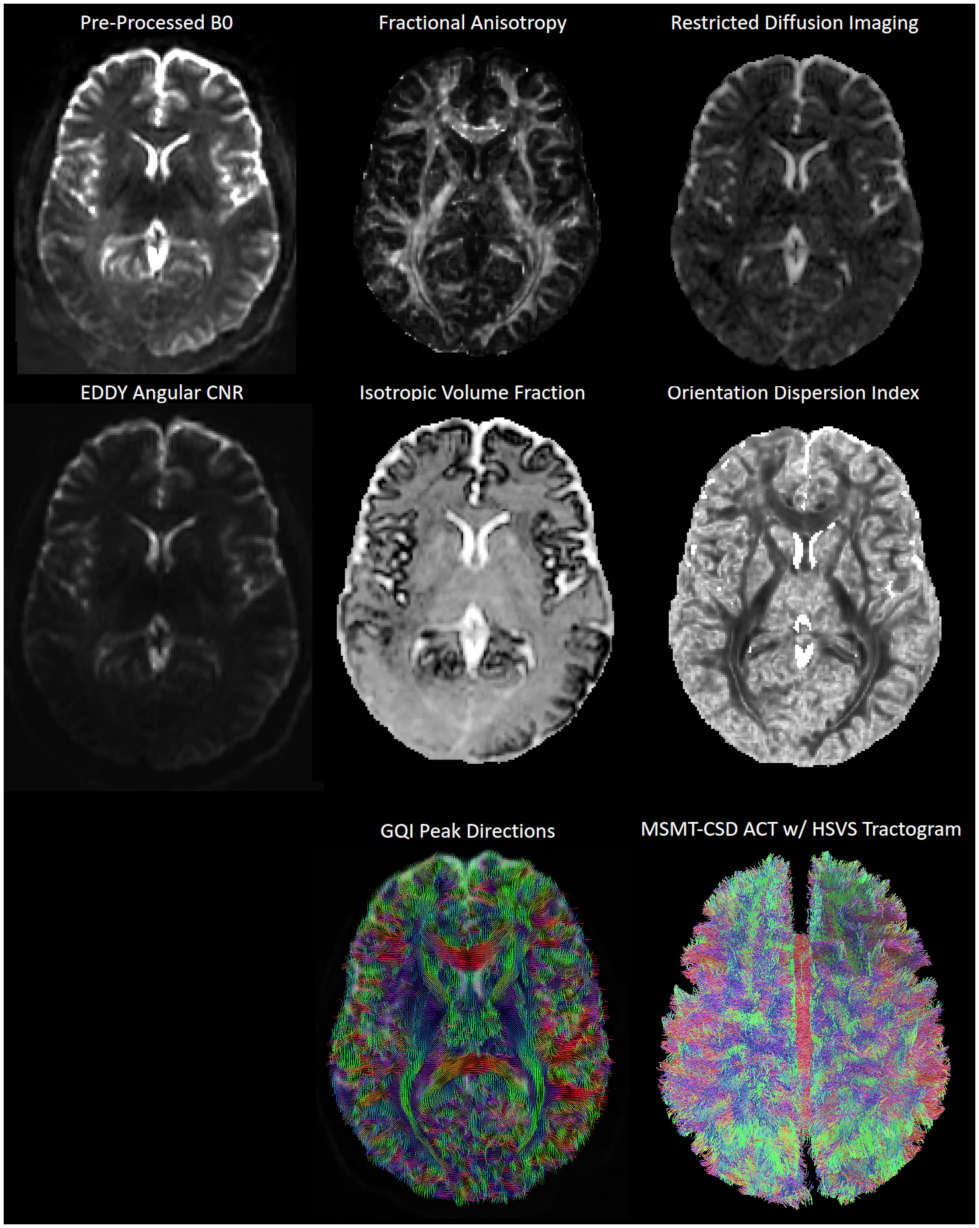
Example diffusion MRI data and outputs from one participant in CUPS. B0: *b*-value 0, CNR: contrast-to-noise ratio (from FSL EDDY), GQI: generalized *q*-sampling imaging, MSMT-CSD ACT w/ HSVS: multi-shell multi-tissue constrained spherical deconvolution reconstructed anatomically constrained tractography with hybrid surface-volume segmentation.

Many internal operations of *QSIPrep* use *Nilearn* 0.8.1 (Abraham et al., 2014, RRID:SCR_001362) and *Dipy* 1.4.1 (Garyfallidis et al., 2014). For more details of the pipeline, see the section corresponding to workflows in *QSIPrep*’s documentation. See Figure 7 for example data.

##### 2.3.5.1 MRtrix3 reconstruction

Multi-tissue fiber response functions will be estimated using the Dhollander algorithm. FODs will be estimated via constrained spherical deconvolution (CSD; Tournier et al., 2004; Tournier et al., 2008)) using an unsupervised multi-tissue method (Dhollander et al., 2019; Dhollander et al., 2016). Reconstructions will be done using Mrtrix3 (Tournier et al., 2019). FODs will be intensity-normalized using mtnormalize (Raffelt et al., 2017).

##### 2.3.5.2 GQI reconstruction

Diffusion orientation distribution functions (ODFs) will be reconstructed using generalized q-sampling imaging (GQI; Yeh et al., 2010) with a ratio of mean diffusion distance of 1.250.

##### 2.3.5.3 NODDI reconstruction

The neurite orientation dispersion and density imaging (NODDI) model (Zhang et al., 2012) will be fit using the AMICO implementation (Daducci et al., 2015). A value of 1.7E-03 will be used for parallel diffusivity and 3.0E-03 for isotropic diffusivity.

#### 2.3.6 Quantitative susceptibility mapping

**Figure 8:**
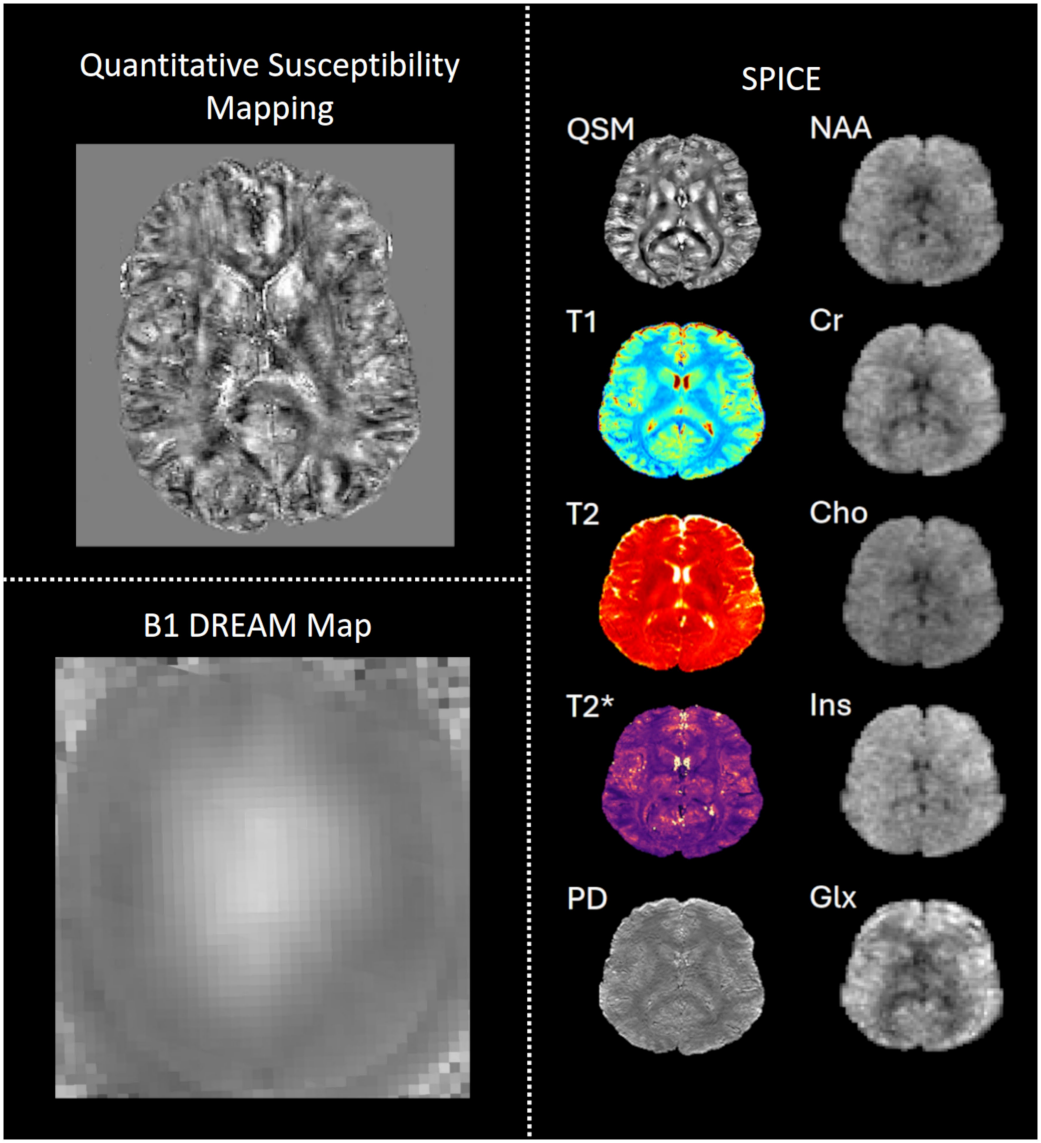
Example quantitative MRI data from one participant in CUPS. SPICE: spectroscopic imaging by exploiting spatiospectral correlation, QSM: quantitative susceptibility mapping, NAA: *N*-acetyl aspartate, Cr: creatine, Cho: choline, Ins: inositol, PD: proton density, Glx: glutamate.

SWI scans will be processed using the 3D GRE workflow in Quantitative Susceptibility Imaging Toolbox (QSMxT; Stewart et al., 2022; Eckstein et al., 2021). Brain masks will be estimated using an Otsu threshold (Otsu, 1975) of ×1.5 for single-pass and ×1.3 for two-pass QSM. Phase unwrapping will use the rapid opensource minimum spanning tree algorithm (ROMEO; Dymerska et al., 2021). Background field removal will beperformed with the projection onto dipole fields method (PDF; Liu et al., 2011b). The rapid two-step dipole inversion method (RTS; Kames et al., 2018) will be used for QSM, yielding a single pass *χ*-map and a two-pass χ-map with automatic artefact reduction (Stewart et al., 2022). See Figure 8 for an example two-pass χ-map. The denoised MP2RAGE images will then be co-registered with the SWI and QSM images using ANTs RegistrationSynQuick (Avants et al., 2009), providing regions of interest from the FreeSurfer Desikan-Killiany atlas parcellation (Desikan et al., 2006).

#### 2.3.7 Hippocampal subfields segmentation

Hippocampal subfields segmentation will be performed on the high-resolution T2*hippocampal images using the Automated Segmentation of Hippocampal Subfields (ASHS) toolbox version 1.0.0 (Yushkevich et al., 2015) and the UMC Utrecht 7 T atlas (Wisse et al., 2016). These will undergo quality control as detailed in (Canada et al., 2023) and are corrected manually as needed. The finalized segmentations will be used to create a hippocampal subfields atlas for 7 T T2* images.

#### 2.3.8 Magnetic resonance elastography reconstruction and processing

**Figure 9:**
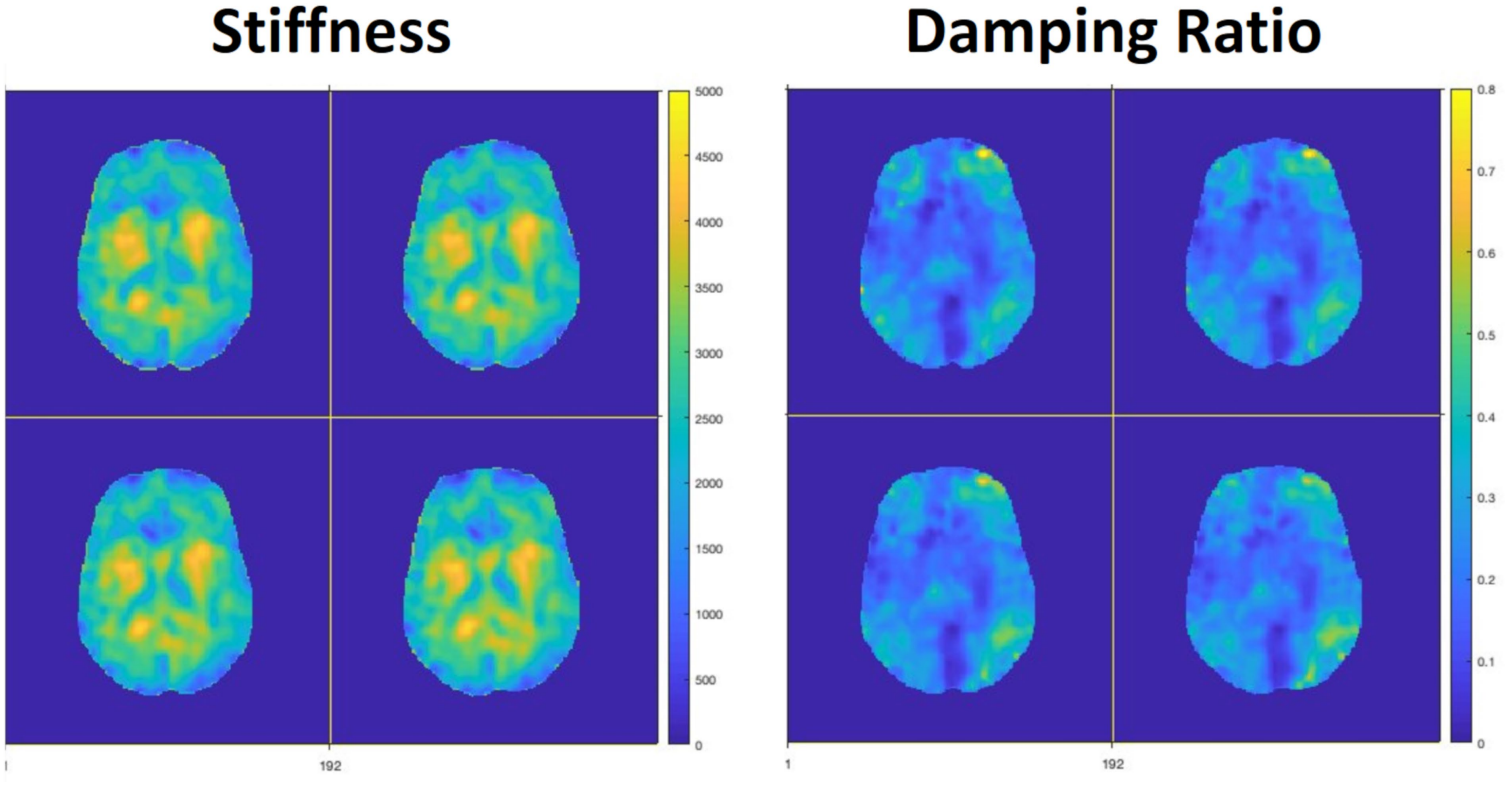
Example MRE data from one participant in CUPS after inversion to mechanical property maps, showing stiffness, μ, in Pascals and damping ratio, *ξ*.

MRE data will be reconstructed through an iterative reconstruction algorithm using our customized high-performance reconstruction platform called PowerGrid (Cerjanic et al., 2016), which incorporates SENSE parallel imaging (Pruessmann et al., 2001), correction for distortions from field inhomogeneity (Sutton et al., 2003), and nonlinear motion-induced phase error correction (Liu et al., 2004; Van et al., 2011). High-resolution reconstructed MRE data will be input into our nonlinear inversion (NLI) algorithm (McGarry et al., 2012; Van Houten et al., 2001; Van Houten et al., 2011) which will return the viscoelastic complex shear modulus, *G* = *G’* + *iG,”* from which we will calculate the stiffness (Manduca et al., 2001), $\mu =2|G|2/(G^{’}+|G|),$ and damping ratio (McGarry and Van Houten, 2008), $\zeta =G^{”}/2G^{’}$ . See Figure 9 for example maps.

#### 2.3.9 Spectroscopic mapping

SPICE data processing will use a MATLAB pipeline. The water-unsuppressed MRSI signals will first be reconstructed from the sparsely sampled signals, through a union-of-subspace model integrated with parallel imaging (Guo et al., 2019). Then the T1 map and T2 map will be generated by linear fitting to the signal equation (Deoni et al., 2003). The B1 inhomogeneity of the water MRSI signals will be corrected using the variable flip angle data (Zhang et al., 2012). The QSM map will be generated from the water MRSI data using HSVD (Barkhuijsen et al., 1987) for field estimation and the Cornell MEDI toolbox for background field removal and QSM dipole inversion (Liu et al., 2011a). To generate metabolite maps, the MRSI data will be pre-processed through field drift correction, eddy current correction, B_0_ field inhomogeneity correction, and water/lipid removal (Ma et al., 2016). Then the spatiospectral functions of MRSI data will be reconstructed from the noisy measurements through a subspace-learning based reconstruction method (Lam et al., 2020; Guo et al., 2025). The metabolite maps will be generated from the reconstructed spatiospectral functions through spectral quantification fitting (Li et al., 2017), with basis functions created from quantum simulation (Soher et al., 2023).

#### 2.3.10 White matter lesion detection

White matter lesion detection will be performed on the T2 FLAIR and MP2RAGE image using Lesion-Mapper-BIDS,^5^ based on the automated script described in Wetter et al. (2016).

#### 2.3.11 Quality control

Quality control metrics will be calculated for MP2RAGE, hippocampal scans, and resting-state fMRI data using *MRIQC* (Esteban et al., 2017). Quality metrics for resting-state processing will be produced by XCP-D (Mehta et al., 2024; Ciric et al., 2017; Parkes et al., 2018). DWI quality metrics will be calculated during preprocessing with QSIPrep (Cieslak et al., 2021). Metrics describing the quality of Freesurfer recon-all will be calculated using the extended python implementation of FSQC (Esteban et al., 2017; Potvin et al., 2016; Reuter et al., 2009; Wachinger et al., 2015).

#### 2.3.12 Face anonymization

Prior to sharing imaging data (e.g.: through OpenNeuro), anatomical NIFTI data will be facially anonymized (Schwarz et al., 2023; Jwa et al., 2024) using *mri_reface* version 0.3.5 (Schwarz et al., 2021) to remove potentially identifiable facial features. Outputs of Freesurfer recon-all that contain facial features will be facially anonymized using the *mideface* tool from Freesurfer v7.4.1.^6^

## 3 Discussion

CUPS uses advanced, quantitative imaging techniques at 7 T to characterize the structural, functional, and biochemical properties in the human brain across a diverse population. The large sample size for 7 T neuroimaging (Hanspach et al., 2021) and broad eligibility criteria will allow the detailed characterization of brain structure and function and their associations with age, physical activity levels, and varying states of health. We acknowledge that the sample size required for age-related effects on some individual modalities (e.g.: resting state fMRI) may be higher than that of this study. The total sample size for this study falls within a range of those of the Human Connectome Project Young Adult 7 Telsa subsample (*n =* 184; Benson et al., 2018), the *n =* 117 recommended by Chu and colleagues to find age-related differences in morphometry for 84 regions of the Freesurfer parcellation at 7 T (Chu et al., 2025), lifespan diffusion MRI studied at 3 T (*n =* 190; Acosta-Franco et al., 2025), and age-related differences in quantitative susceptibility mapping values in subcortical regions at 3 T (*n =* 55; Howard et al., 2022). We aim to contribute to the current body of 7 T neuroimaging data through the combination of modalities included in this study. The quality control results from the CUPS study will be available to expand the normative 7 T image quality metrics for future studies. A hippocampal subfield atlas will be produced using high in-plane resolution T2*-weighted images. Uniquely, the availability of raw k-space data will enable the development and testing of advanced image reconstruction and analysis procedures. Moreover, the publication of this 7 T dataset will allow investigators worldwide to examine related questions of interest.

## Footnotes

1
https://cupopulationstudy.illinois.edu/participate/

2
https://nihtoolbox.org/get-the-toolbox/

3
https://xcp-d.readthedocs.io

4
https://qsiprep.readthedocs.io/en/latest/workflows.html

5
https://github.com/mrfil/lesion-mapper-bids

6
https://surfer.nmr.mgh.harvard.edu/fswiki/MiDeFace
